# Anti-leukemic effects of the V-ATPase inhibitor Archazolid A

**DOI:** 10.18632/oncotarget.6180

**Published:** 2015-10-19

**Authors:** Siwei Zhang, Lina S. Schneider, Binje Vick, Michaela Grunert, Irmela Jeremias, Dirk Menche, Rolf Müller, Angelika M. Vollmar, Johanna Liebl

**Affiliations:** ^1^ Department of Pharmacy, Pharmaceutical Biology, Ludwig-Maximilians-University, Munich, Germany; ^2^ Department of Gene Vectors, Helmholtz Zentrum München, German Research Center for Environmental Health, Munich, Germany; ^3^ Department of Oncology/Hematology, Dr. von Haunersches Kinderspital, Munich, Germany; ^4^ German Cancer Consortium (DKTK), German Cancer Research Center (DKFZ), Heidelberg, Germany; ^5^ Kekulé-Institut für Organische Chemie und Biochemie der Universität Bonn, Bonn, Germany; ^6^ Helmholtz Institute for Pharmaceutical Research Saarland, Helmholtz Centre for Infection Research and Department of Pharmaceutical Biotechnology, Saarland University, Saarbrücken, Germany

**Keywords:** Archazolid, leukemia, natural compounds

## Abstract

Prognosis for patients suffering from T-ALL is still very poor and new strategies for T-ALL treatment are urgently needed. Our study shows potent anti-leukemic effects of the myxobacterial V-ATPase inhibitor Archazolid A. Archazolid A reduced growth and potently induced death of leukemic cell lines and human leukemic samples. By inhibiting lysosomal acidification, Archazolid A blocked activation of the Notch pathway, however, this was not the mechanism of V-ATPase inhibition relevant for cell death induction. In fact, V-ATPase inhibition by Archazolid A decreased the anti-apoptotic protein survivin. As underlying mode of action, this work is in line with recent studies from our group demonstrating that Archazolid A induced S-phase cell cycle arrest by interfering with the iron metabolism in leukemic cells. Our study provides evidence for V-ATPase inhibition as a potential new therapeutic option for T-ALL.

## INTRODUCTION

T-cell acute lymphoblastic leukemia (T-ALL) is an aggressive hematopoietic malignancy. Despite advances in understanding of the molecular basis of T-ALL and intensified treatment regimens that have improved outcome of therapy, some patients fail conventional chemotherapy and T-ALL remains fatal in 20% of children and more than 50% of adults [1]. Along this line, new therapeutic approaches are urgently needed to improve patient prognosis.

Activating mutations in Notch1 occur in more than 50% of T-ALLs, highlighting Notch1 as key player in T-ALL [2, 3]. In fact, constitutive activation of Notch1 signaling represents the most prominent oncogenic pathway in T-cell transformation [1]. Therefore, targeted therapies to inhibit the Notch1 pathway have been developed. γ-secretase inhibitors (GSIs) that prevent Notch1 activating cleavage have been widely tested in preclinical models and clinical trials. However, the role of Notch in leukemia is still not entirely clear: on the one hand, it was shown that Notch signaling promotes cell survival and apoptosis resistance in leukemia [4, 5] and that Notch blockade by γ-secretase inhibition exerts pro-apoptotic effects in leukemia [6]. On the other hand, activation of Notch or of the Notch downstream target Hes1 inhibits leukemia development, growth and survival [7-9]. Furthermore, GSIs have shown only modest and temporary responses in leukemia therapy. These controversial results about Notch in leukemia demand for better understanding of the mechanisms that contribute to Notch signaling in leukemia in order to develop novel strategies to inhibit Notch1 signaling with alternative mechanism different from GSIs as potential promising approaches for T-ALL therapy.

Besides Notch activation at the plasma membrane where ligand binding initiates y-secretase dependent cleavage of the Notch receptor, and subsequently NICD generation and translocation to the nucleus, Notch signaling in part depends on endocytosis. Recent reports showed that Notch can be activated in endosomes: in *drosophila*, Notch signaling is abolished when endosomal entry is blocked but is enhanced when endosomal retention is increased [10]. Moreover, in *drosophila*, acidification of endosomes by the V-ATPase is required for Notch activation [11].

Along this line, we hypothesized that inhibition of V-ATPase might be a promising strategy for T-ALL treatment. V-ATPase is an ATP-dependent proton pump that regulates pH homeostasis by translocating protons across membranes. The main function of V-ATPase is to regulate the acidification of intracellular compartments like lysosomes [12, 13]. V-ATPases are multisubunit heteromeric protein complexes with two functional domains: the V1 domain that is composed of eight subunits (A-H) mediates ATP hydrolysis and the V0 domain that is composed of five subunits (a, c, c’’, d, e) regulates proton translocation [14]. The V-ATPase is localized at endosomes and lysosomes and is essential for endocytotic processes, receptor internalization and recycling, and finally lysosomal degradation [13]. Therefore, V-ATPase is implicated in fundamental physiological processes, like neurotransmitter uptake, renal acidification, bone resorption or sperm maturation and is associated with pathologic conditions including osteopetrosis, renal tubular acidosis and disease-related processes such as entry of toxins and viruses [13].

Recent reports indicate important functions of V-ATPase in tumors. Augmented expression of V-ATPase in cancer cells was demonstrated to contribute to metastasis, survival and growth of cancer cells [15-18]. Localization of V-ATPase at the plasma membrane was associated with invasiveness of various types of cancer including breast, pancreatic, prostate, and melanoma cancer cells [14, 19-21]. V-ATPase was shown to localize to the plasma membrane in sarcoma cells as well and was elucidated as a promising target for Ewing sarcoma, osteosarcoma, chondrosarcoma or rhabdomyosarcoma [22, 23] as well as glioblastoma [24, 25]. Along this line, V-ATPase inhibition could represent a promising approach for tumor therapy. Although only few V-ATPase inhibitors have been described so far, their V-ATPase binding properties and mode of action are well-investigated. Amongst others, a class of natural compounds - the Archazolids - inhibits V-ATPase activity by binding to the V-ATPase V0 subunit [26-28] that is responsible for proton transport across the membrane [29]. Archazolids are macrolides that have originally been isolated from the myxobacterium *Archangium gephyra* [30], and are available also by chemical synthesis [31, 32]. Archazolids have attracted attention as highly potent V-ATPase inhibitors that exert promising anti-tumor effects [15-18, 33-36].

Because Notch signaling activation in part depends on endocytosis [10, 11, 37] and V-ATPase has therefore been linked with the Notch pathway [35, 38], we hypothesized that V-ATPase inhibition might represent an alternative option to target leukemic cells. Therefore, we had a closer look on the functional effects and the mechanism of action, including the Notch pathway and cellular stress response, of the V-ATPase inhibitor Archazolid A in leukemic cells.

## RESULTS

### V-ATPase in leukemic cells

**Figure 1:**
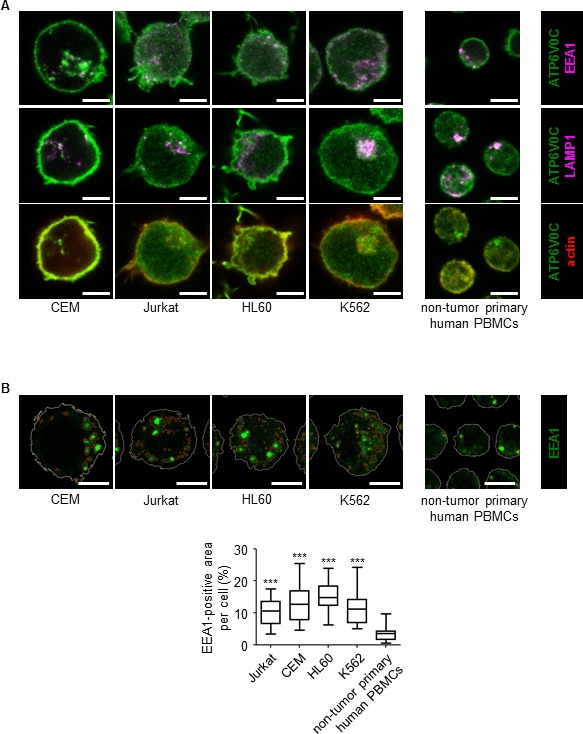
V-ATPase in leukemic cell lines **A.** V-ATPase localization in leukemic cell lines is shown. Immunostaining of non-tumor primary human peripheral blood mononuclear cells (PBMCs) and leukemic cell lines (Jurkat, CEM, HL60, K562) for V-ATPase c-subunit (ATP6V0C, green) together with EEA1 (magenta), LAMP1 (magenta) and actin (red) is shown. Scale bar 5 μm. **B.** The size of the endo-lysosomal compartment in leukemic cell lines is shown. Staining of non-tumor primary human PBMCs and leukemic cell lines (Jurkat, CEM, HL60, K562) for EEA1 (green) is shown. Scale bar 5 μm. The white line labels the cell border. The red lines label the endosome area (EEA1-positive area). Endosome area per cell was calculated and is shown in the graph. One-Way ANOVA, Tukey's post test, ***p ≤ 0.001 (compared to non-tumor primary PBMCs).

First, we analyzed the expression of the V-ATPase subunits in different leukemic cell lines including the T-ALL cell lines Jurkat and CEM, the AML cell line HL60, and the CML cell line K562 in comparison to non-tumor primary human PBMCs. Most of the V-ATPase subunits were comparably expressed in non-tumor PBMCs, Jurkat, CEM, and HL60 cells and some subunits were increased in K562 cells (Table 1). Immunostainings show that subunit c ATP6V0C which is targeted by Archazolid A, is localized to the lysosomal system and to the plasma membrane of leukemic cells (Figure 1A). As V-ATPase is essential for endo-lysosomal function, we analyzed the size of the endo-lysosomal compartment. In fact, the size of the endosomal compartment was increased in leukemic cell lines compared to non-tumor primary human peripheral blood mononuclear cells (PBMCs) (Figure 1B). This set of data suggests a potential function of V-ATPase in leukemia.

**Table 1:** mRNA expression of V-ATPase subunits of the V1 domain (A-H) and the V0 domain (a, c, c’’, d, e) is shown in human leukemic cell lines related to non-tumor primary human PBMCs

V-ATPase subunit	S-Jurkat	CEM	K562	HL60
ATP6V1A	0.70±0.07	1.41±0.11	2.83±0.47	0.70±0.05
ATP6V1B1	0.43±0.16	1.16±0.60	1.24±1.13	0.36±0.19
ATP6V1B2	0.52±0.07	0.63±0.10	0.88±0.10	0.68±0.12
ATP6V1C1	0.90±0.06	0.93±0.09	2.59±0.22	0.83±0.09
ATP6V1C2	0.79±0.21	0.48±0.11	0.75±0.15	34.28±15.71
ATP6V1D	0.96±0.01	1.19±0.14	1.87±0.05	0.24±0.01
ATP6V1E1	1.00±0.05	0.97±0.01	3.43±0.44	0.89±0.06
ATP6V1E2	0.51±0.05	0.37±0.03	0.65±0.06	0.17±0.01
ATP6V1F	1.12±0.27	1.64±0.34	5.27±0.39	1.00±0.06
ATP6V1G1	1.00±0.17	1.06±0.06	1.61±0.13	0.60±0.04
ATP6V1G2	0.12±0.03	1.00±0.14	0.11±0.03	0.06±0.01
ATP6V1G3	0.18±0.07	0.69±0.25	0.10±0.00	0.11±0.04
ATP6V1H	0.85±0.04	1.16±0.06	2.32±0.08	0.66±0.04
ATP6V0A1	0.48±0.01	1.07±0.06	10.67±0.87	0.60±0.05
ATP6V0A2	0.78±0.03	0.66±0.21	1.55±0.10	0.30±0.04
ATP6V0A3	0.01±0.00	0.11±0.01	0.37±0.24	0.68±0.07
ATP6V0A4	0.82±0.31	1.46±0.23	1.26±0.71	0.83±0.28
ATP6V0B	1.53±0.12	1.47±0.14	4.78±0.53	1.46±0.49
ATP6V0C	0.67±0.06	0.81±0.07	2.39±0.36	0.90±0.10
ATP6V0D1	0.38±0.03	0.44±0.04	1.26±0.12	0.86±0.09
ATP6V0D2	0.10±0.04	0.19±0.17	9.09±4.84	18.38±5.61
ATP6V0E1	0.34±0.08	0.72±0.16	0.84±0.03	0.62±0.04
ATP6V0E2	1.97±0.08	2.62±0.15	0.54±0.05	0.07±0.01

### V-ATPase inhibition by Archazolid A impairs growth and induces death of leukemic cell lines

**Figure 2:**
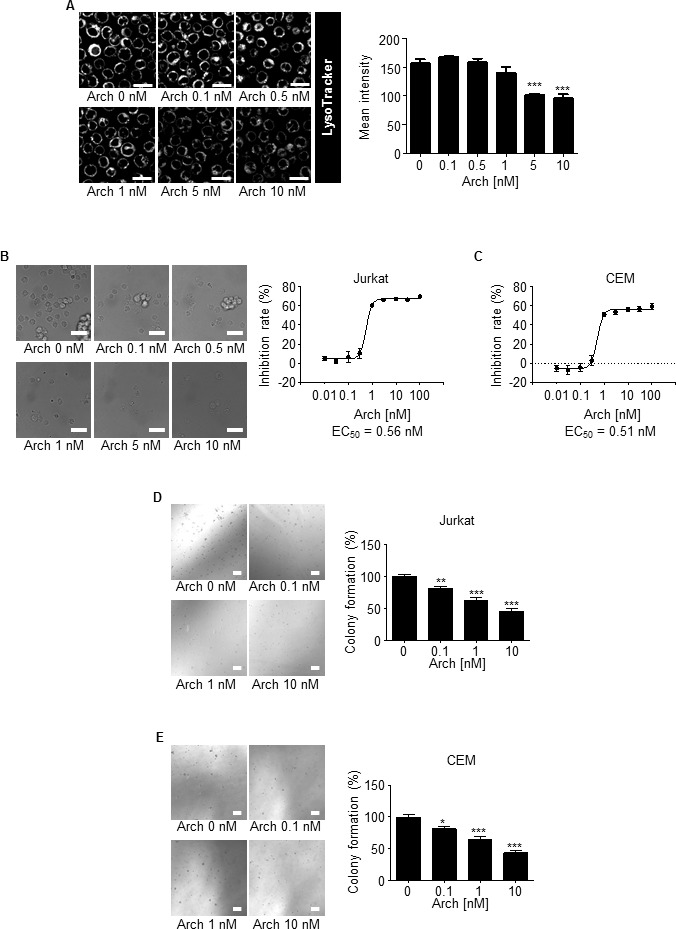
Archazolid A inhibits growth of leukemic cell lines **A.** Archazolid A inhibits lysosome acidification. Stainings of Jurkat cells treated with Archazolid A (Arch, 0, 0.1, 0.5, 1, 5, 10 nM, 24h) with the pH-sensitive LysoTracker are shown. *n* = 3. Scale bar 20 μm. Quantification of LysoTracker staining is displayed (***p ≤ 0.001, One-Way ANOVA, Tukey, *n* = 3). **B.**, **C.** Archazolid A inhibits the proliferation of leukemic cells. Inhibition rates of proliferation of Jurkat **B.** and CEM cells **C.** after treatments with Archazolid A (Arch) at indicated concentrations for 72h are shown. EC50 is indicated. *n* = 3. Scale bar 50 μm. **D.**, **E.** Archazolid A inhibits clonogenic growth. Colony formation of Jurkat **D.** and CEM cells **E.** after treatments with Archazolid A (Arch) at indicated concentrations is shown. Scale bar 100 μm. One-Way ANOVA, Tukey's post test, *p ≤ 0.05, **p ≤ 0.01, ***p ≤ 0.001, *n* = 3.

Archazolid A inhibited V-ATPase activity in leukemic cells as shown by staining of lysosomes with a pH-sensitive fluorescence dye (LysoTracker) (Figure 2A). Archazolid A impaired proliferation of leukemic cell lines Jurkat (EC50 0.56 nM) and CEM (EC50 0.51 nM) (Figure 2B, 2C). In line, clonogenic growth of Jurkat and CEM cells was reduced by V-ATPase inhibition (Figure 2D, 2E).

**Figure 3:**
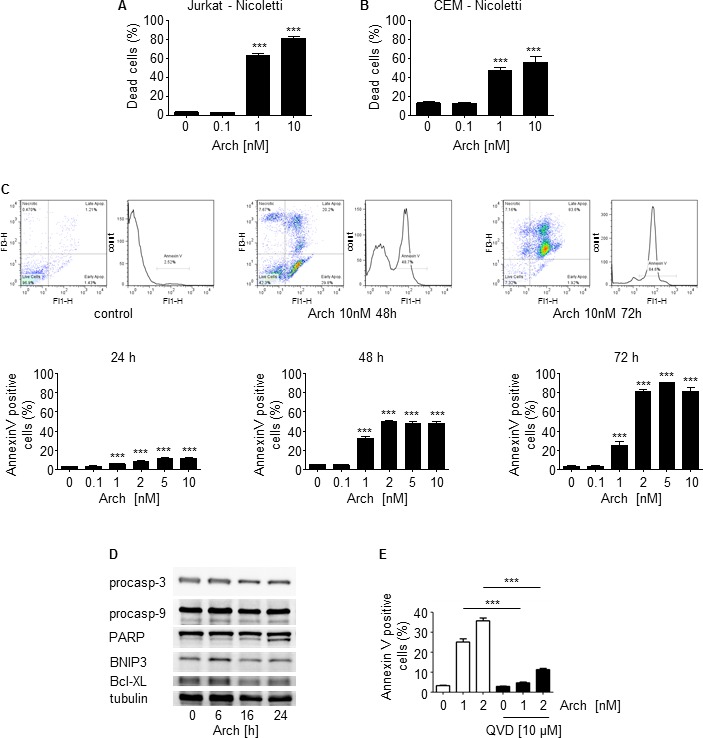
Archazolid A induces death of leukemic cell lines **A.**, **B.** Apoptosis rate determined by Nicoletti assay of Jurkat **A.** and CEM cells **B.** after treatments with Archazolid A (Arch) at indicated concentrations for 72h is shown. One-Way ANOVA, Tukey's post test, ***p ≤ 0.001, *n* = 3. **C.** Pictures display Annexin V staining of Jurkat cells after treatments with Archazolid A (Arch). Bar graphs show the apoptosis rate determined by Annexin V staining of Jurkat cells after treatments with Archazolid A (Arch) at indicated concentrations for 24h, 48h, and 72h. One-Way ANOVA, Tukey's post test, ***p ≤ 0.001, *n* = 3. **D.** Immunoblots of Jurkat cells treated with Archazolid A (10 nM) for the indicated times for procaspase-3 (procasp-3), procaspase-9 (procasp-9), PARP, BNIP3, and Bcl-XL are shown. The immunoblot for tubulin indicates equal loading. *n* = 3. **E.** Annexin V/PI staining of cells treated with Archazolid A at indicated concentrations for 48h and with/without the pan-caspase inhibitor QVD-OPh (QVD) at 10 μM for 48h is shown. One-Way ANOVA, Tukey's post test, ***p ≤ 0.001, *n* = 3.

Moreover, as shown by Nicoletti assay (Figure 3A, 3B) and Annexin V staining (Figure 3C), Archazolid A potently induced death of leukemic cell lines. In line with a previous report from our group [15], Archazolid A induced cleavage of procaspase-3, procaspase-9, and PARP, increased the pro-apoptotic protein BNIP3, and decreased the anti-apoptotic protein Bcl-XL in leukemic cells (Figure 3D). Moreover, the pan-caspase inhibitor QVC-OPh partially rescued Archazolid A induced apoptosis (Figure 3E). This suggests that apoptosis by Archazolid A is at least partially mediated via the mitochondrial pathway.

### V-ATPase inhibitor Archazolid A induces death of primary human leukemic cells

**Figure 4:**
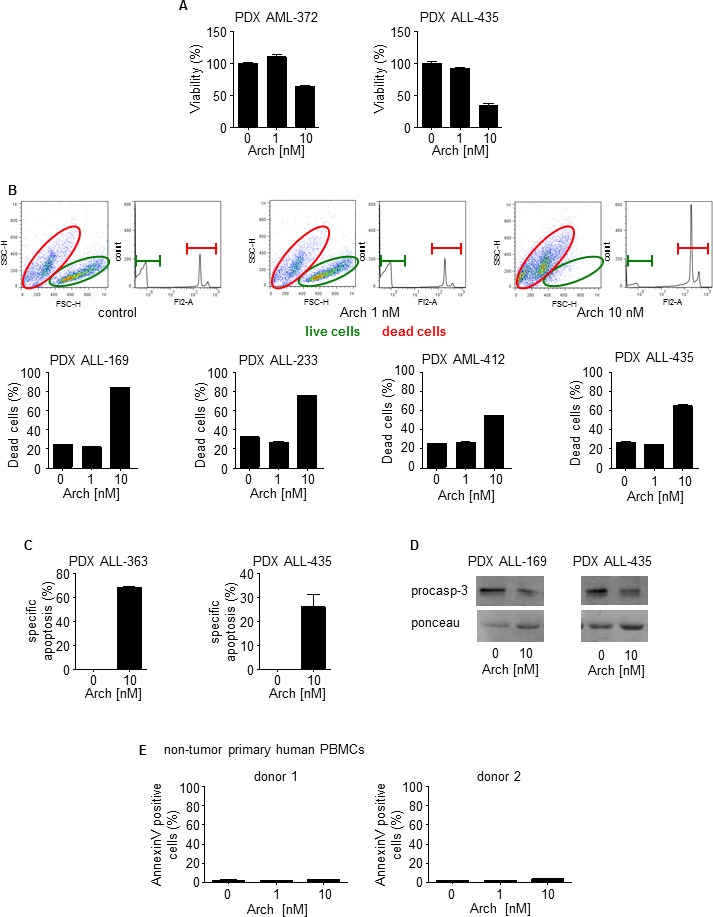
Archazolid A induces cell death in human patient derived xenograft (PDX) samples **A.** Viability of leukemic PDX samples with/without treatment with Archazolid A (Arch) for 72h at indicated concentrations is shown. **B.** PI exclusion staining of leukemic PDX samples with/without treatment with Archazolid A (Arch) for 48h at indicated concentrations is shown. Upper panels display dot plots and histograms of PDX leukemic cells from one respective patient (PDX ALL-169). Dead cells are stained by PI and are marked in red. Live cells without PI staining are displayed in green. Lower panels show apoptosis rate of PDX leukemic cells treated with Archazolid A (Arch) at indicated concentrations. **C.** The specific apoptosis rate determined by Annexin V/PI staining of PDX cells after treatments with Archazolid A (Arch) at indicated concentrations for 48h is shown. **D.** Immunoblots of PDX samples treated with Archazolid A (10 nM, 48h) for procaspase-3 (procasp-3) are shown. Ponceau staining indicates equal loading. **E.** Archazolid A does not induce cell death in non-tumor primary human PBMCs. Apoptosis rate determined by Annexin V/PI staining and of non-tumor primary human PBMCs (FACS analysis with gating for lymphocytes) of two different donors treated with Archazolid A (Arch) at indicated concentrations for 48h is shown.

To analyze the potential therapeutic relevance of V-ATPase inhibition by Archazolid A, patient derived xenograft (PDX) leukemic cells were studied. Clinical characteristics are listed in Table 2. PDX cells enable repetitive *in vitro* testing on viable patient-derived cells, by passaging primary tumor cells in severely immuno-compromised mice. These cells resemble the primary patient cells to a very high extent [39, 40]. In accordance with cell culture experiments, Archazolid A reduced viability (Figure 4A) and induced death of PDX human leukemic cell samples from different patients which was again shown by PI exclusion assays (Figure 4B) and Annexin V staining (Figure 4C). Moreover, Archazolid A induced cleavage of procaspase-3 in PDX samples (Figure 4D). This set of data demonstrates that V-ATPase inhibition by Archazolid A exerts anti-leukemic properties. Because Archazolid A did not induce cell death of non-tumor primary human PBMCs (Figure 4E) it might represent an option for anti-leukemic treatment.

**Table 2:** Clinical characteristics of patients from which the PDX cells have been generated are shown

number	Type of Leukemia	Disease stage	sex	age	cytogenetics
ALL-169	cALL	diagnosis	m	pediatric	unknown
ALL-233	pre B ALL	diagnosis	m	pediatric	t(2;15)(p13;q15)
ALL-256	cALL	unknown		pediatric	+8, t(9;22)(q34;q11)
ALL-363	pre B ALL	diagnosis	m	adult	BCR/ABL translocation
AML-372	AML	relapse	m	adult	Complex, including −17
AML-412	AML	diagnosis	f	adult	CN, FLT3-ITD, NPM1+
ALL-435	pre B ALL	unknown		pediatric	t(11;19)

### V-ATPase inhibition by Archazolid A addresses Notch1 signaling in leukemic cells, but the Notch pathway is not responsible for Archazolid A induced cell death

**Figure 5:**
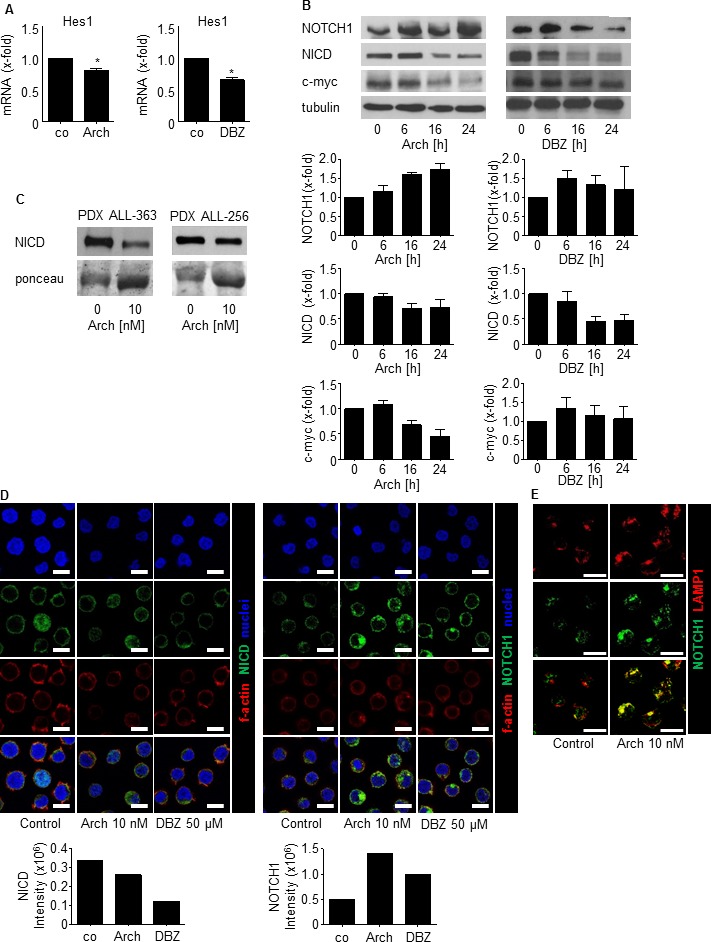
Archazolid A inhibits Notch1 signaling **A.** Hes1 mRNA expression of Jurkat cells treated with Archazolid A (Arch, 10 nM, 24h) or DBZ (50 μM, 24h) is shown. Archazolid A: paired *t*-test, *p = 0.0341, *n* = 3. DBZ: paired *t*-test, *p = 0.0090, *n* = 3. **B.** Immunoblots from Jurkat cells treated with Archazolid A (Arch, 10 nM, left panel) or DBZ (10 μM, right panel) for the indicated times and probed with antibodies for Notch, NICD, and c-myc are shown. Immunoblots for β-tubulin indicate equal loading. Bar graphs display quantitative evaluations of immunoblots for Notch1, NICD, and c-myc. *n* = 3. **C.** Immunoblots from PDX cells treated with Archazolid A (10 nM, 24h) and probed with antibodies for NICD are shown. Ponceau staining is used as loading control. **D.** Immunostainings from Jurkat cells treated with Archazolid A (Arch, 10 nM, 24h) or DBZ (50 μM, 24h) for NICD (green, left panels) and Notch1 (green, right panels) are shown. *n* = 3. Scale bar 10 μm. Bar graphs display quantitative evaluations of NICD and Notch1 intensities. **E.** Immunostainings from Jurkat cells treated with Archazolid A (Arch, 10 nM, 24h) for LAMP1 (red) and Notch1 (green) are shown. Merged pictures indicate colocalization of LAMP1 and Notch1 (yellow). *n* = 3. Scale bar 10 μm.

In order to evaluate the mechanism of Archazolid A to induce leukemic cell death, the Notch pathway gained our attention. Expression of the Notch1 downstream target Hes1 was reduced by Archazolid A as well as by the y-secretase inhibitor (GSI) Dibenzazepine (DBZ) that served as positive control for Notch signaling inhibition (Figure 5A). Archazolid A reduced levels of Notch1 intracellular domain (NICD) and the Notch1 downstream target c-myc (Figure 5B). As expected, DBZ treatment decreased NICD and c-myc as well. NICD expression revealed that the Notch1 pathway was active in PDX samples (Figure 5C). Archazolid A-mediated decrease of NICD (Figure 5C) proved that Archazolid A addressed the Notch1 pathway in PDX cells. Archazolid A induced cell death in these PDX samples as well (Figure 4C and 10A), suggesting sensitivity of leukemic cells with Notch1 pathway activation towards Archazolid A. Moreover, Archazolid A increased levels of the Notch1 whole receptor (Figure 5B). This was further analyzed by immunostainings which demonstrate again that Archazolid A abrogated NICD levels whereas the Notch1 whole receptor was increased (Figure 5D). Stainings for the lysosomal marker LAMP1 revealed large lysosomes that contained increased amounts of Notch1 in Archazolid A treated cells (Figure 5E). This suggested that Archazolid A inhibits Notch1 signaling in a way different from GSI: by capturing the Notch1 whole receptor in lysosomes and therefore inhibiting Notch1 cleavage and NICD generation at endolysosomal membranes.

**Figure 6:**
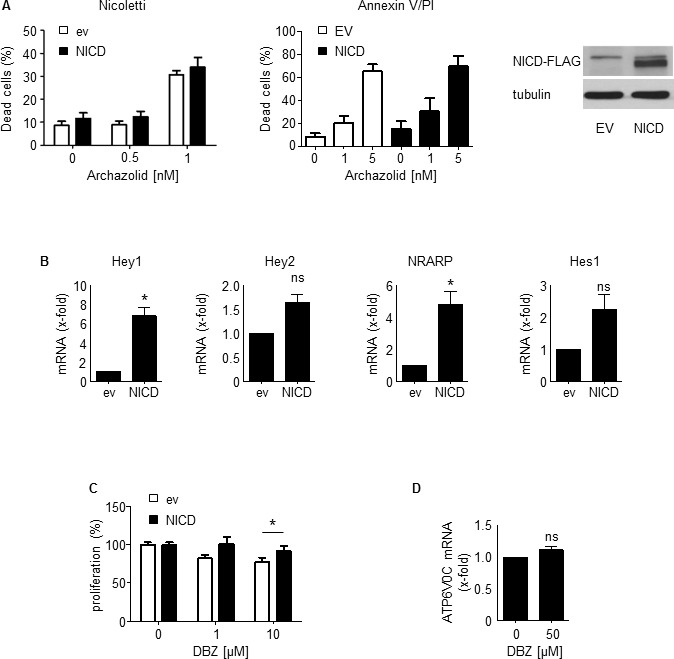
NICD cannot rescue Archazolid A mediated induction of apoptosis **A.** Apoptosis rate determined by Nicoletti assay (left bar graph) and by Annexin V/PI staining (right bar graph) of Jurkat cells overexpressing either empty vector or NICD after treatment with Archazolid A (48h) is shown. Immunoblots show NICD overexpression. *n* = 3. **B.** Increased expression of Notch downstream targets Hey1 (paired *t*-test, *p = 0.0197), Hey2 (paired *t*-test, *p* = 0.0576), NRARP (paired *t*-test, *p = 0.0407), Hes1 (paired *t*-test, *p* = 0.1117) after NICD overexpression (24h) is shown. *n* = 3. **C.** Proliferation of Jurkat cells overexpressing either empty vector or NICD after treatment with DBZ at indicated concentrations for 72h is shown. *t*-test, *p = 0.0209, *n* = 3. **D.** Expression of V-ATPase subunit c (ATP6V0C) of Jurkat cells treated with DBZ (50 μM, 24h) is shown. Non-significant (ns), paired *t*-test, *p* = 0.1886.

However, NICD overexpression could not rescue from Archazolid A-mediated cell death (Figure 6A), suggesting no causal relationship between the Notch1 pathway and Archazolid A-induced leukemic cell death. Nevertheless, NICD overexpression induced Notch1 downstream target gene expression (Figure 6B) and rescued DBZ-induced inhibition of proliferation (Figure 6C), proving that NICD overexpression was functional. In order to better understand the inter-connection between V-ATPase and Notch1, we analyzed whether Notch inhibition affects levels of V-ATPase c-subunit. V-ATPase c-subunit expression was not affected by DBZ treatment (Figure 6D). This set of data indicated that Notch1 signaling inhibition is not the major relevant downstream signaling of Archazolid A in leukemic cell death induction.

### Inhibition of y-secretase impairs growth but does not induce death of leukemic cell lines

**Figure 7:**
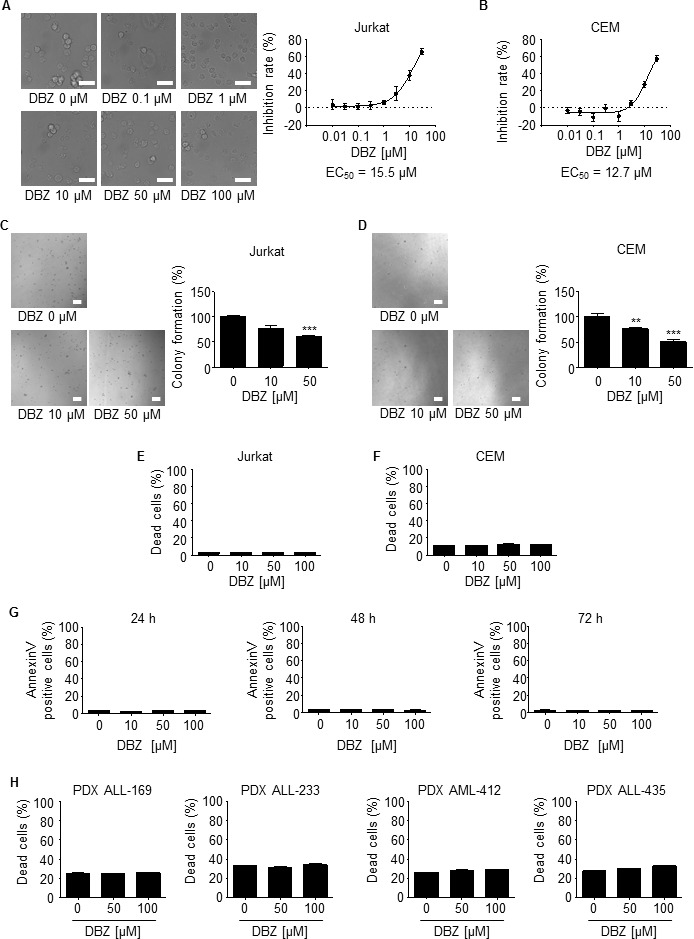
y-secretase inhibition by DBZ inhibits growth but does not induce leukemic cell death **A.**, **B.** DBZ inhibits proliferation of leukemic cells. Inhibition rates of proliferation of Jurkat **A.** and CEM cells **B.** after treatment with DBZ for 72h at indicated concentrations are shown. EC50 is indicated. *n* = 3. Scale bar 50 μm. **C.**, **D.** Colony formation of Jurkat **C.** and CEM cells **D.** after treatments with DBZ at indicated concentrations is shown. Scale bar 100 μm. One-Way ANOVA, Tukey, **p ≤ 0.01, ***p ≤ 0.001, *n* = 3. **E.**, **F.** Apoptosis rate determined by Nicoletti assay of Jurkat **E.** and CEM cells **F.** after treatments with DBZ at indicated concentrations for 72h is shown. *n* = 3. **G.** Apoptosis rate determined by Annexin V/PI staining of Jurkat cells after treatments with DBZ at indicated concentrations for 24h, 48h, and 72h is shown. 24h and 48h: *n* = 2. 72h: *n* = 3. **H.** Apoptosis rate determined by PI exclusion of PDX leukemic cells with/without treatments with DBZ (48h) at indicated concentrations is shown.

DBZ-mediated γ-secretase inhibition reduced proliferation of leukemic cell lines Jurkat (EC50 15.5 μM) and CEM (EC50 12.7 μM) (Figure 7A, 7B) as well as clonogenic growth (Figure 7C, 7D). However, DBZ neither induced death of leukemic cell lines (Figure 7E-7G), nor of human leukemic PDX samples (Figure 7H). This set of data suggests that the Notch1 pathway is not essential for the effects of Archazolid A on leukemic cell death.

### Archazolid A decreases survivin by inducing cell cycle arrest and interfering with the iron metabolism in leukemic cells

**Figure 8:**
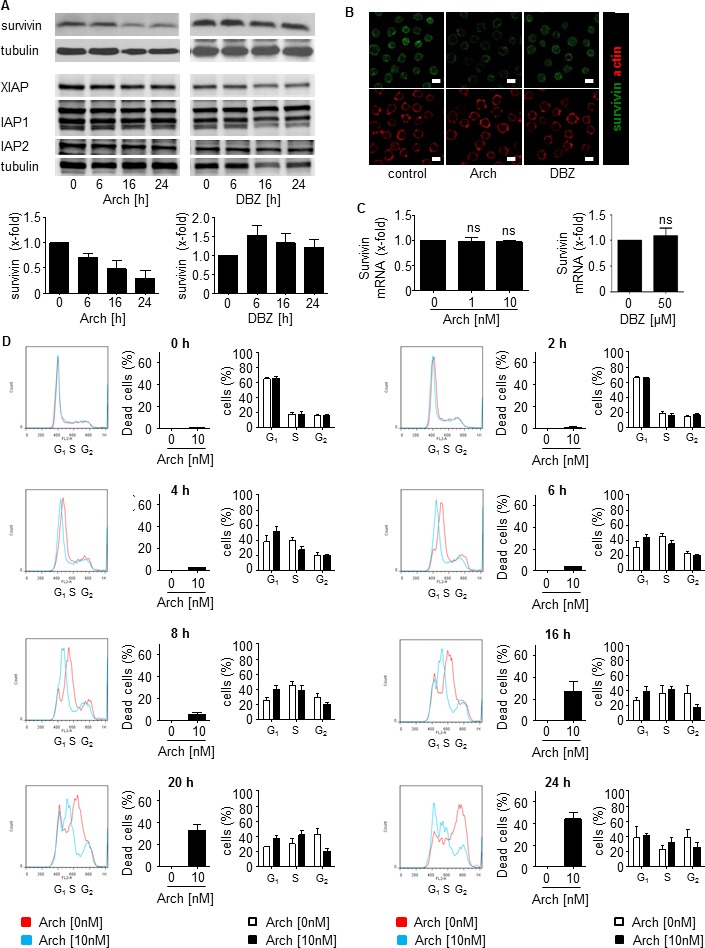
Archazolid A decreases the anti-apoptotic protein survivin and interferes with the cell cycle in leukemic cells **A.** Archazolid A decreases the anti-apoptotic protein survivin. Immunoblots from Jurkat cells treated with Archazolid A (Arch, 10 nM, left panel) or DBZ (50 μM, right panel) for the indicated times and probed with antibodies for survivin, XIAP, IAP1, and IAP2 are shown. Immunoblots for tubulin indicate equal loading. *n* = 3. Bar graphs display the quantitative evaluation of survivin expression. **B.** Immunostainings for survivin (green) and actin (red) after treatment with/without Archazolid A (Arch, 10 nM, 24h) and DBZ (50 μM, 24h) is shown. Scale bar 10 μm. **C.** Archazolid A (Arch) and DBZ do not interfere with survivin mRNA expression. Survivin mRNA levels from Jurkat cells treated with Archazolid A (1 and 10 nM) and DBZ (50 μM) for 24h are shown. Not significant (ns), Archazolid A: One-Way ANOVA, DBZ: paired *t*-test, *n* = 3. **D.** Archazolid A (Arch) induces S-phase cell cycle arrest of Jurkat cells. Cell cyle analysis and apoptosis measurement after aphidicolin synchronization (24h) and subsequent treatment with Archazolid A for indicated times is shown. Control cells (untreated, Archazolid A 0 nM) are indicated in red, Archazolid A (Arch, 10 nM) treated cells are indicated in blue. One representative out of three independent experiments is shown.

In search for other pathways of Archazolid A responsible for cell death, we analyzed the anti-apoptotic protein survivin, a crucial regulator of cell death in leukemic cells [41-43]. In fact, Archazolid A decreased survivin protein levels (Figure 8A, 8B and Supplementary Figure 1) which was not due to changed survivin mRNA (Figure 8C). In contrast, DBZ showed no significant effect on survivin (Figure 8A-8C), again providing evidence that Archazolid A-mediated anti-leukemic effects are not based on Notch1 inhibition. Because survivin expression strongly depends on the cell cycle [41-43], subsequently, cell cycle analysis was performed. In fact, cells without Archazolid A treatment pass S-phase (8h) to reach (16h) and accumulate (20h, 24h) in G2-phase. In contrast, Archazolid A treated cells accumulate in S-phase (8h, 16h, 20h) but do not traverse to G2-phase. This suggests that Archazolid A treated cells die during S-phase which is confirmed by cell death analysis showing that Archazolid A-induced S-phase arrest is in parallel with apoptosis induction (Figure 8D).

**Figure 9:**
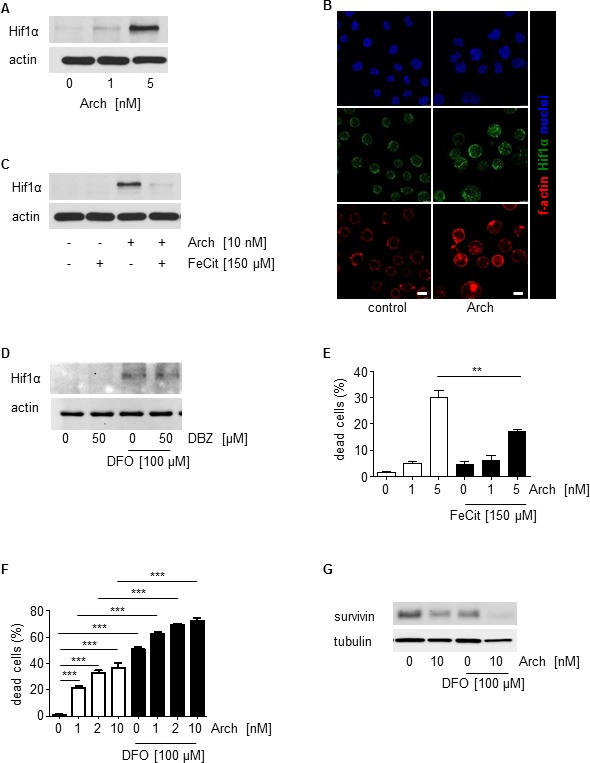
Archazolid A interferes with the iron metabolism in leukemic cells **A.** Archazolid A increases Hif1α. Immunoblots show Hif1α levels of Jurkat cells with/without Archazolid A (Arch) treatment at indicated concentrations for 24h. Actin indicates equal loading. **B.** Immunostainings for Hif1α (green) and f-actin (red) after treatment with/without Archazolid A (Arch, 10 nM, 24h) is shown. Nuclei are labeled with Hoechst33342 (blue). Scale bar 7.5 μm. **C.** Archazolid A mediated Hif1α increase is abrogated by iron citrate. Immunoblots show Hif1α levels of Jurkat cells with/without Archazolid A (Arch) and iron citrate (FeCit) treatment at indicated concentrations for 24h. Actin indicates equal loading. *n* = 3. **D.** Inhibition of Notch by DBZ does not influence Hif1α. Immunoblots of Jurkat cells treated with DBZ and deferoxamine (DFO) at indicated concentrations for 24h for Hif1α and actin (loading control) are shown. **E.** Archazolid A mediated cell death is partially rescued by iron citrate. The graph shows cell death of Jurkat cells treated with/without Archazolid A (Arch) and iron citrate (FeCit) at indicated concentrations for 48 h. Mann Whitney test, **p = 0.0022, *n* = 3. **F.** DFO induces cell death in Jurkat cells and is enhanced by Archazolid A. Nicoletti assay of cells treated with/without Archazolid A (Arch) and DFO at indicated concentrations for 48 h is shown. One-Way ANOVA, Tukey's post test, ***p ≤ 0.001, *n* = 3. **G.** Survivin is decreased by DFO which is enhanced by Archazolid A. Immunoblots for survivin and tubulin (loading control) from cells treated with/without DFO (100 μM) and Archazolid A (Arch, 10 nM) for 48h are shown; *n* = 3.

In line with recent studies from our group [15, 44] that elucidated that interference of Archazolid with the iron metabolism leads to S-phase cell cycle arrest in breast cancer, Archazolid A stabilized Hif1α in leukemic cells (Figure 9A-9C) which was abrogated by simultaneous treatment with iron citrate (Figure 9C). In contrast, Notch inhibition by DBZ did not affect Hif1α (Figure 9D). As shown previously [44], Archazolid A-mediated cell death was partially rescued by iron citrate (Figure 9E). Because Hif1α stabilization is generally pro-proliferative, but also anti-proliferative and cell death inducing properties have been described [45-48], we analyzed the effects of Hif1α induction on cell death and survivin expression in leukemic cells. Induction of Hif1α by deferoxamine (DFO) induced leukemic cell death and decreased survivin, which was even enhanced by concomitant Archazolid A treatment (Figure 9F, 9G). This set of data suggests that interference of Archazolid A with the iron metabolism contributes to cell death in leukemic cells.

**Figure 10:**
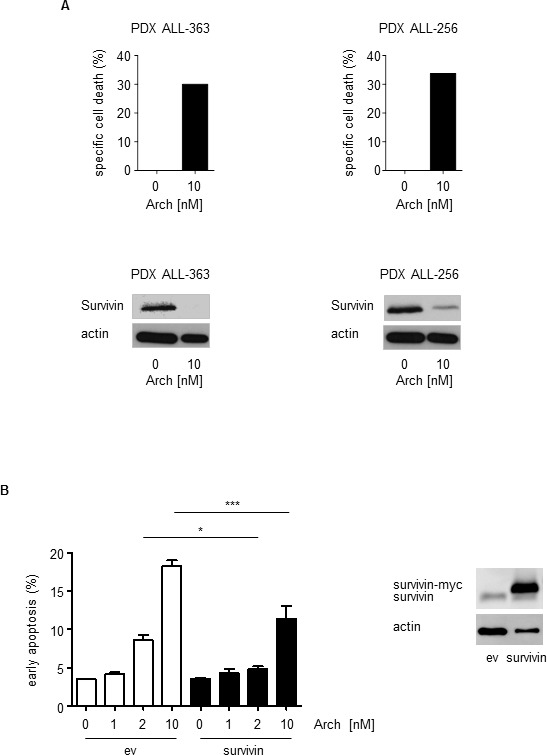
Archazolid A-induced apoptosis in PDX is in line with decreased levels of the anti-apoptotic protein survivin **A.** Upper panels show apoptosis rates (specific cell death) determined by PI exclusion staining of PDX leukemia samples treated with Archazolid A (Arch, 10 nM, 48 h). Lower panels display immunoblots from PDX cells from the same patients treated with Archazolid A (Arch, 10 nM, 24 h) and probed with antibodies for survivin. Immunoblots for actin indicate equal loading. **B.** Archazolid A mediated cell death is partially rescued by survivin overexpression. The graph shows early apoptosis (AnnexinV-positive and PI-negative cells) determined by AnnexinV/PI staining of Jurkat cells overexpressing either empty vector (ev) or survivin and treated with/without Archazolid A (Arch) at indicated concentrations for 48 h. One-Way ANOVA, Tukey, *p ≤ 0.05, ***p ≤ 0.001, *n* = 3. Immunoblots show overexpression of empty vector (ev) and survivin 24h after transfection; actin indicates equal loading.

Finally, coincident with apoptosis induction, survivin levels were decreased by Archazolid A treatment of PDX human patient samples as shown for the PDX samples PDX ALL-363 and PDX ALL-256 (Figure 10A, Figure 4C). Moreover, survivin overexpression partially rescued Archazolid A induced cell death in leukemic cells (Figure 10B). This set of data suggests decreased survivin as relevant mechanism of Archazolid A to induce leukemic cell death.

## DISCUSSION

Our study demonstrates that V-ATPase inhibition by Archazolid A exerts anti-leukemic effects, suggesting V-ATPase inhibition as potential option for T-ALL treatment.

During recent years, V-ATPase has emerged as interesting target for cancer therapy. Amongst others, including various studies of our group, it has been shown that V-ATPase is implicated in cancer cell metastasis [16, 49], invasion [50, 51], tumor cell death [15, 52], anoikis resistance [17], cellular stress response [15], breast cancer trastuzumab resistance [34], and regulation of the secretion profile of cancer and cancer-associated cells [18, 53]. Only few reports point to a function of V-ATPase in leukemia or hematopoietic cells in general. In detail, it was shown that proton pump inhibitors induce apoptosis of human B-cells [54] and lysosome disruption has been associated with anti-leukemic effects in acute myeloid leukemia (AML) [55]. Moreover, V-ATPase was suggested to regulate leukemic cell adhesion [56] and V-ATPase inhibition by Bafilomycin A reduced leukemic cell growth [35].

In search for the signaling pathway responsible for the anti-leukemic effects of Archazolid A-mediated V-ATPase inhibition, first the Notch pathway was analyzed as it is one of the most prominent oncogenic pathways in T-ALL [57]. Targeted therapies addressing the Notch pathway have been proposed as auspicious options for T-ALL treatment. Despite promising *in vitro* results and initial clinical effectiveness of GSIs that block Notch activatory cleavage, this benefit only in some studies translates into improved overall survival [58-60]. In fact, most human T-ALL cell lines are resistant to GSIs that fail to induce leukemic cell death [57]. Therefore, to inhibit Notch signaling with a mechanism different from GSIs might be a promising approach. Our study shows that V-ATPase reduces Notch1 signaling by capturing the Notch whole receptor in the endolysosomal compartment and inhibiting its activatory cleavage. This is in line with reports demonstrating that impaired endolysosomal function by V-ATPase inhibition blocks γ-secretase mediated Notch activatory cleavage at the endolysosomal membrane [11, 35, 37, 38]. However, our rescue experiments revealed that leukemic cell death by Archazolid A was not caused by inhibition of Notch signaling. Moreover, although our results revealed that GSI treatment reduced leukemic cell growth, cell death was not induced. Thus, Archazolid A-mediated induction of leukemic cell death was not based on Notch1 pathway inhibition.

Our work suggests the anti-apoptotic protein survivin as target addressed by Archazolid A to induce leukemic cell death. Survivin exerts multiple cellular functions: it participates in the regulation of cell division, apoptosis, stress response, migration, and metastasis. Whereas it is nearly absent in normal tissues, it is overexpressed in most human tumors, including hematopoietic malignancies [42, 43, 61-63]. Survivin expression has been associated with leukemia progression and poor clinical outcome [64-66]. In consequence, survivin is addressed in cancer therapy by molecular antagonists like antisense oligonucleotides, siRNA, or hammerhead ribozymes, as well as small molecules or cancer immunotherapeutics [67]. Inhibition of survivin has shown clinical benefits and chemosensitizing effects in leukemia [42, 43, 68-70]. By describing potent anti-leukemic effects of Archazolid A, our study probably expands the class of small molecule survivin antagonists.

Survivin expression strongly depends on the cell cycle, i.e. survivin is upregulated and stabilized during G2-phase [41]. Besides regulation at the transcriptional level, changes in protein stability essentially contribute to survivin expression during the cell cycle: proteasome-dependent destruction of survivin has been shown in interphase cells whereas at metaphase, mitotic phosphorylation of survivin by Cdk1 has been associated with increased protein stability [71]. In fact, in line with recent studies from our group addressing V-ATPase in cancer [15, 44], our results presented here suggest that Archazolid decreased survivin at the protein level by inducing S-phase cell cycle arrest in leukemic cells which probably was due to its interference with the iron metabolism.

In line with previous studies from our group showing that Archazolid B led to cellular stress response and induction of Hif1α [15, 44], our present study suggests that the induction of Hif1α contributes to Archazolid A induced leukemic cell death. Although the stabilization of Hif1α during hypoxia is generally pro-proliferative as stabilization of Hif1α influences the survival of tumor cells, Hif1α has also tumor-inhibiting properties. In detail, Hif1α overexpression inhibits cell proliferation by expressing cell cycle inhibitors like p53, p21, and p27 and promotes apoptosis by inducing pro-apoptotic molecules such as p53, Nip3, and Noxa [45-48].

A recent study from our group demonstrated that inhibition of V-ATPase by Archazolid B led to iron depletion by disruption of transferrin receptor recycling which subsequently reduced ribonucleotide reductase (RNR) activity, induced S-phase cell cycle arrest and finally caused cell death [44]. Interestingly, iron overload due to inefficient erythropoiesis or blood transfusion represents a major problem in patients suffering from myelodysplastic syndromes (MDS), clonal disorders with ineffective hematopoiesis and an increased risk of transformation into acute myeloid leukemia. Iron overload is associated with osteopenia and osteoporosis in these patients [72, 73]. In consequence, V-ATPase inhibition in hematopoietic diseases might be an interesting object for further studies.

To conclude, our study provides evidence for V-ATPase inhibition as new alternative strategy and Archazolid A as interesting new compound for T-ALL therapy.

## MATERIALS AND METHODS

### Cells

Cell lines: Human leukemia Jurkat T cells (J16, S-Jurkat) were kindly provided by P.H. Krammer and H. Walczak, Heidelberg, Germany. S-Jurkat cells were cultured in RPMI 1640 (PAN Biotech, Aidenbach, Germany) containing 10% FCS (PAA Laboratories, Cölbe, Germany) and 1% pyruvate (Merck, Darmstadt, Germany). CCRF-CEM cells were kindly provided by Dr. Joachim Arend (Mainz, Germany) and cultured in RPMI 1640 containing 10% FCS.

### Reagents

The γ-secretase inhibitor DBZ was purchased from Merck Millipore. The pan-caspase inhibitor QVD-OPh (551476) was purchased from Calbiochem. Deferoxamine (DFO) was purchased from Sigma.

### Patient-derived xenograft (PDX) leukemic cells

The xenograft mouse model, the transplantation of patient's primary tumor cells, and the cell amplification have been described previously [39, 40]. In detail, primary leukemic cells were obtained from diagnostic bone marrow aspiration or peripheral blood sampling before onset of treatment. Xenografts were established by injecting 10^7^ fresh or frozen/thawed primary leukemic cells into NSG mice (NOD SCID gamma mice, i.e. mice with dysfunctional gamma chain of the IL-2R receptor) by tail vein injection. Development of leukemia was monitored by repetitive blood sampling in mice and by determining human leukemic cells in flow cytometry upon staining of murine CD45 and human CD38. At clear leukemic engraftment or by latest after 25 weeks, mice were sacrificed and human patient-derived xenograft (PDX) leukemic cells were isolated either from bone marrow or from spleens of mice using Ficoll purification. Phenotypic markers and genetic aberrations in PDX cells compared to primary leukemic tumor cells remained mainly stable as shown before [25]. For stimulation and during experiments, cells were maintained in RPMI 1640 medium containing 20% FCS and 1% glutamine. Freshly isolated PDX leukemic cells were stimulated as indicated *in vitro*.

### Primary non-tumor human peripheral blood mononuclear cells (PBMCs)

Isolation of primary non-tumor human PBMCs was performed using the “Ficoll-Paque PLUS” kit from GE Healthcare according to the manufacturer's instructions. Briefly, EDTA (1.5mg/ml) blood (2 ml) was mixed with balanced salt solution (2 ml), added on top of Ficoll-Paque PLUS (3ml) in a 15ml centrifuge tube, and centrifuged (400×g, 40min, RT). Afterwards, PBMCs were carefully withdrawn from the interface between the upper plasma layer and the lower Ficoll-Paque PLUS layer, washed twice by 3 volumes of balanced salt solution, centifuged (100×g, 10 min, RT) and resuspended in culture medium (RPMI 1640+20%FCS+1%Glutamine). PBMCs were directly used for the respective assays.

For FACS analysis, gating of lymphocytes was performed and only these cells were included in the evaluation.

### Tansfection of cells

Cell transfection was performed by using the Amaxxa system with the cell line nucleofector kit V (Lonza, VCA-1003) according to the manufacturer's instructions. 1×10^6^ cells were electroporated (program X-001). NICD plasmid was from addgene (20183). Survivin plasmid was from Sino Biologicals (pCMV3-BIRC5-myc, HG10356-CM G09AU4M62). For respective assays, cells were used 24h (NICD) or 48h (survivin) after transfection.

### Proliferation

CellTiter-Blue^®^ assay was performed according manufacturer's instructions. Briefly, cells were seeded (96 well plates, 3×10^3^ cells/well) for 24 h, treated with Archazolid A or DBZ at indicated concentrations and incubated for 70 h. CTB solution (20μl) was added and cells were incubated for 2h. Fluorescence was measured with a Tecan reader (Maennedorf, Austria).

### PDX viability

PDX leukemic cells were seeded (96 well plates, 1×10^5^ cells/well) and the cells were treated with Archazolid A at indicated concentrations for 72h. Cell Titer Blue assay was performed according manufacturer's instructions as described before at “proliferation”. For calculating viability, the value from the time of plating was subtracted.

### Apoptosis and cell cycle

Apoptosis and cell cycle analysis were performed according to the Nicoletti method [74]. In brief, cells were seeded (24 well plates, 0.5×10^4^ cells/well for 48 h, 2.5×10^4^ cells/well for 72 h) and treated with Archazolid A or DBZ at indicated concentrations. For DNA staining, cells were permeabilized and stained by resuspension in hypotonic fluorochrome solution (HFS) containing propidium iodide (PI, 50 μg/ml) and incubated at 4°C overnight. Subsequently, flow cytometry was performed on a FACSCalibur (Becton Dickinson, Heidelberg, Germany). The sub-G1 peak accounts for apoptotic cells and was determined according to Nicoletti [74]. Cell cycle was analyzed by using FlowJo 7.6 (Tree Star Unc., Ashland, USA).

### PI exclusion

Cells were seeded (96 well plates, 1×10^5^ cells/well and treated with Archazolid A or DBZ at indicated concentrations. Subsequently, cells were incubated with PI (5 μg/ml, 5 min) that was added to the cell suspension before analysis by flow cytometry. Subsequently, flow cytometry was performed on a FACSCalibur (Becton Dickinson, Heidelberg, Germany) using the Fl-2 laser.

As cells were not permeabilized, PI staining was exclusive for dead cells, whereas viable cells were not stained by PI. For quantification, PI positive dead cells can be seen in dot plots at the left and in histograms at the right and are shown in red; PI negative viable cells can be seen in dot plots at the right and in histograms at the left and are shown in green (Figure 4A).

### Annexin V/PI staining

Annexin V/PI staining was performed by using the Annexin V-FITC Apoptosis Detection Kit (eBioscience Dx, BMS500FI/300CE) according to the manufacturer's protocol. Briefly, cells were treated as indicated, collected, centrifuged (600g, 4°C, 10min), washed with PBS, centrifuged again (600g, 4°C, 10min) and resuspended in binding buffer. 5μl AnnexinV-FITC were added to 195μl of cell suspension, cells were mixed and incubated at RT for 10 min (cover from light), centrifuged (600g, 4°C, 10min), washed with 200μl Binding Buffer and resuspended in Binding Buffer. 10μl PI (20μg/ml) was added to 190 μl of cell suspension and FACS analysis was performed using Fl-2 and Fl-3 lasers. AnnexinV positive cells have been considered as apoptotic. AnnexinV positive and PI negative cells have been considered as early apoptotic.

For primary PDX leukemic cells, the specific apoptosis was calculated according to the following formula: % specific apoptosis = 100 × (% total apoptosis of treated cells - % total apoptosis of untreated cells) / (100 - % total apoptosis untreated cells) [75].

For primary non-tumor human PBMCs, gating of lymphocytes was performed and only these cells were included in the evaluation.

### Colony formation

Cells were seeded (6 well plates, 5×10^5^ cells/well) and treated with Archazolid A or DBZ at indicated concentrations for 24h. Cells were freshly plated (96 well plates, 5×10^3^ cells/well) in RPMI 1640 Medium containing 40% FCS, 0.52% Methylcellulose, and 1% Sodium Pyruvate and incubated for 11 d. Images from each well were taken with a Zeiss 510 Meta Confocal Microscope. Colonies were counted by using Image J with the cell counter plugin.

### Immunoblotting

Immunoblotting was performed as described [76]. The following primary antibodies were used: actin (MAB1501 Millipore), BCL-XL (2762 Cell Signaling), BNIP3 (ab10433 Abcam), Caspase 3 (sc-7148 Santa Cruz), Caspase 9 (9506 Cell Signaling), Hif1α (610958 BD), IAP-1 (4952 Cell Signaling), IAP-2 (3130 Cell Signaling), NICD (4147 Cell Signaling), Notch1 (4380 Cell Signaling), c-Myc (sc-788 Santa Cruz), survivin (2803 Cell Signaling), β-tubulin (2164 Cell Signaling), XIAP (610717 BD).

### RT-PCR

mRNA was isolated using the Qiagen RNeasy Mini Kit. For reverse transcription the High-Capacity cDNA Reverse Transcription Kit (Applied Biosystems) was used. RT-PCR was performed with the 7300 Real Time PCR System.

For RT-PCR of Notch downstream targets, the following Taqman gene expression assays were used: Hes1 Hs00172878, HEY1 Hs00232618, HEY2 Hs00232622, NRARP Hs01104102 (Life Technologies Corporation, Carlsbad, CA, USA). GAPDH was used as housekeeper.

For RT-PCR for V-ATPase subunits, SYBR® Green PCR Master Mix (4309155, Life Technologies) was used. A list with the primer sequences for all V-ATPase subunits is included in Supplementary Table 1.

### Confocal microscopy

For Immunostaining with antibodies, cells were treated as indicated, collected, resuspended and seeded on chrome alum-gelatin (0.05% CrK(SO_4_)_2_·12H_2_O, 0.4% gelatine in H_2_O) coated coverslips (30 min). Cells were fixed (4% PFA, 10mins), permablized (0.2% Triton X 100 in PBS, 5min), blocked (0.2% BSA in PBS, 1 h), incubated with primary antibodies (0.2% BSA in PBS, 1h) and subsequently secondary antibodies (0.2% BSA in PBS, 1h) and mounted. The following primary antibodies were used: EEA1 (sc-6415, Santa Cruz), LAMP1 (H4A3, Developmental Studies Hybridoma Bank), NICD (4147 Cell Signalling), Notch1 (4308, Cell Signaling). Alexa Fluor conjugated secondary antibodies (Molecular Probes) were used.

For LysoTracker experiment, cells were treated as indicated, collected, and stained with LysoTracker (Molecular Probes) for 45min. After Hoechst33342 staining (5 μg/ml, 5min), confocal microscopy was performed with a Zeiss LSM 510 META confocal microscope.

For the evaluation of the size of the endolysosomal compartment, EEA1 staining was performed. The size of the endolysosomal compartment was analyzed by calculating the EEA1-positive area per cell by using ImageJ. Approximately 20 cells per cell line were randomly selected for the evaluation.

The quantification of the intensities of Notch1 and NICD staining was performed by using Image J. The total intensity of the whole image was divided by the number of cells. Cells on the border of the images were excluded.

### Statistic evaluation

All experiments were performed at least 3 times in duplicates/triplicates. Results are expressed as mean value ± SEM. One-way ANOVA/Tukey and individual t-tests were conducted using GraphPad Prism (version 5.04, GraphPad Software, Inc.). P values less than 0.05 were considered as significant.

## SUPPLEMENTARY MATERIAL FIGURE AND TABLE


