# Supplementary material for: Anti-leukemic effects of the V-ATPase inhibitor Archazolid A
Source: Oncotarget. 2015 Oct 19;6(41):43508–28. doi: 10.18632/oncotarget.6180 (PMC4791247; doi:10.18632/oncotarget.6180)
Supplement: Supplementary file 1 [file oncotarget-06-43508-s001.pdf]

Anti-leukemic effects of the V-ATPase inhibitor Archazolid A

Supplementary Material

Supplementary Figure 1

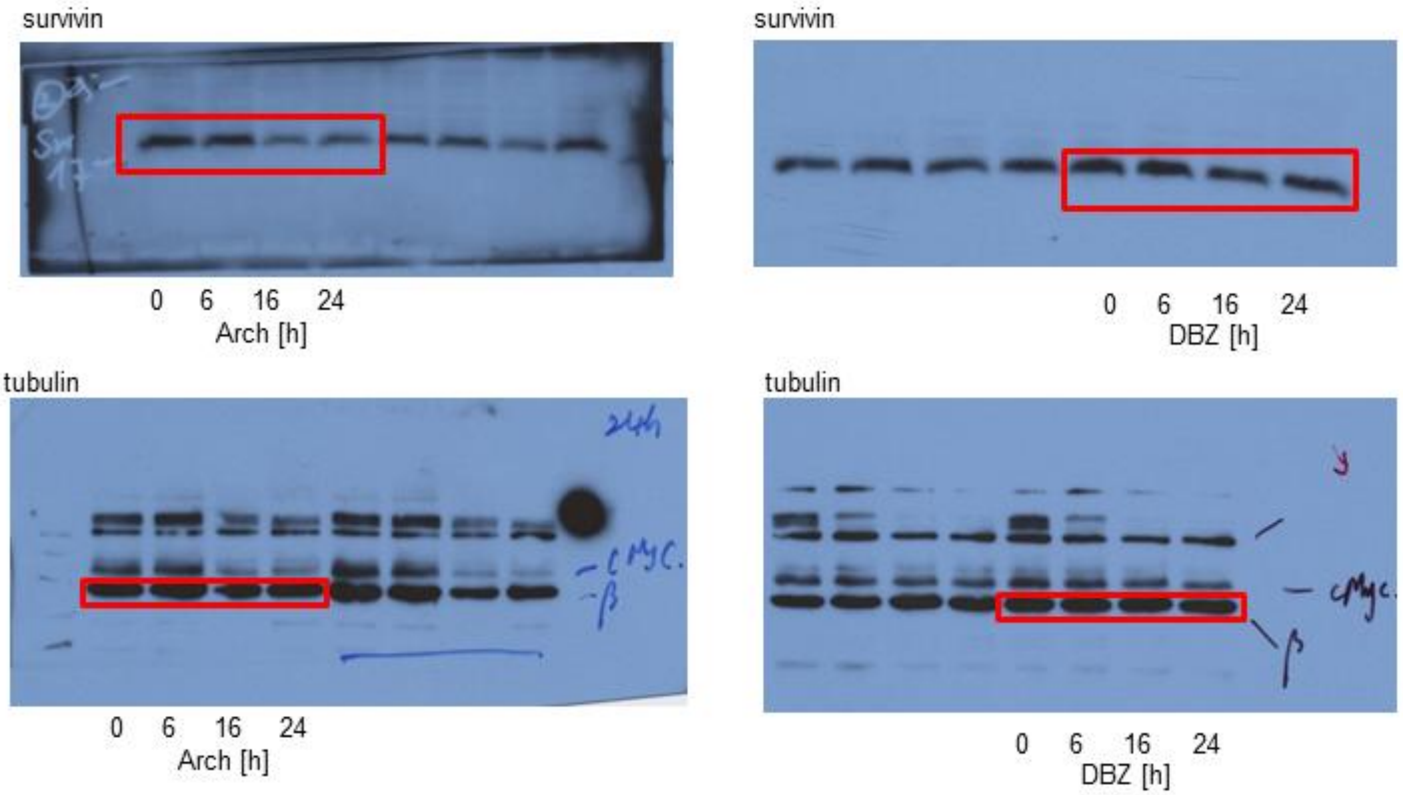

Full blots related to Fig. 8A are shown. Immunoblots from Jurkat cells treated with Archazolid A (Arch, 10 nM, 24h, left panel) or DBZ (50 µM, 24h, right panel) and probed with antibodies for survivin are shown. Immunoblots for tubulin indicate equal loading.

Supplementary Table 1: RT-PCR Primers used for V-ATPase subunits

Gene Name		Sequence (5'->3')
ATP6V1A	Forward	GATGTCTGAAGTCCTCCGGG
	Reverse	TGGCTACCAAAGCTGTCCTC
ATP6V1B1	Forward	ACAGATCCCCATCCTCACCA
	Reverse	TGTGAAGCTGTCTGTCCACG
ATP6V1B2	Forward	CCAGGAAGGATCATGCCGAT
	Reverse	GGGCTTCTTCTCCAACGACA
ATP6V1C1	Forward	GCATGCGGCAACTTCAAAGA
	Reverse	GCCAACCAAGACATCCAACG
ATP6V1C2	Forward	GGAAACTCGACACCTTTGCTG
	Reverse	AAGTCAACTCCGTTTGCCAG
ATP6V1D	Forward	CGCAAGTGAAGATTCGAGCG
	Reverse	TTCCCCACCTCTGGCTAAAC
ATP6V1E1	Forward	TTTCCCTCTGGTAAAGGCTGC
	Reverse	CAGCTATGTCTTCAGGCAGGT
ATP6V1E2	Forward	AGAGGCATACCTGGCTGTGA
	Reverse	GAGATCCAGTCGGCTTTCCA
ATP6V1F	Forward	CCTCATCAACCAGTACATCGCA
	Reverse	CGTCATATGGGTGCTCCTTGG
ATP6V1G1	Forward	GGCTAGTCAGTCTCAGGGGA
	Reverse	CCGGTTCTTTCTTTTGCGGG
ATP6V1G2	Forward	AGCAGAGAAACCGAGAGCG
	Reverse	GGCAGAAATCCGGTAGTTGGG
ATP6V1G3	Forward	GCCAAGGAGGAAGCAATGGT
	Reverse	TCTGAGAGCCCATTATCTTAGATTG
ATP6V1H	Forward	TGCAAACAAAGTCAACTGGCA
	Reverse	GCTTCTCTTCAGGGCTTCGT
ATP6V0A1	Forward	ATGGACACCGGTGAAAACCC
	Reverse	CAGAGCTTCCTGGTTTGTGTTG
ATP6V0A2	Forward	CTGGGATTTGTGTCTGGCCT
	Reverse	CACGATGGTGTACCCTTTGC
ATP6V0A3	Forward	CCTACACCTGCGTGAGTCG
	Reverse	AACCACAAAGCGTCTCTGGA
ATP6V0A4	Forward	TGACCGGAAAGTTGGGGTTC
	Reverse	AGTACACGTTTCCTCGGCAG
ATP6V0B	Forward	ACTTCGCCCTTCATGTGGTC
	Reverse	CAATGATGGAGGAGCCGGTAA
ATP6V0C	Forward	ATGCTTCGTTTTTCGCCGTC
	Reverse	ATGACAGACATGGCCGCAA
ATP6V0D1	Forward	AGATCATCCGCAACACCCTC
	Reverse	CACATGGCATCAGCCGTAGT
ATP6V0D2	Forward	CCAGATGAAGAACGTAGCGGA
	Reverse	ACGCTCGTAAAACACGTCCT
ATP6V0E1	Forward	GGTGCCTTGGTTCATCCCTAA
	Reverse	GGTTGAGTTGGGCCAGAATTG
ATP6V0E2	Forward	CATTCGCCCTCCCGGTCAT
	Reverse	TCACTCCGCGGTTGGGTC
